# Aerobic Biodegradation Characteristic of Different Water-Soluble Azo Dyes

**DOI:** 10.3390/ijerph15010035

**Published:** 2017-12-26

**Authors:** Shixiong Sheng, Bo Liu, Xiangyu Hou, Bing Wu, Fang Yao, Xinchun Ding, Lin Huang

**Affiliations:** State Key Laboratory of Pollution Control and Resource Reuse, School of the Environment, Nanjing University, Nanjing 210046, China; raffaello7@126.com (S.S.); houxy145@163.com (X.H.); bwu@nju.edu.cn (B.W.); yaof1992@hotmail.com (F.Y.); 13951010830@126.com (X.D.); hlin2001@126.com (L.H.)

**Keywords:** azo dye, aerobic biodegradation, Acid Orange 7, Sudan I, solubility

## Abstract

This study investigated the biodegradation performance and characteristics of Sudan I and Acid Orange 7 (AO7) to improve the biological dye removal efficiency in wastewater and optimize the treatment process. The dyes with different water-solubility and similar molecular structure were biologically treated under aerobic condition in parallel continuous-flow mixed stirred reactors. The biophase analysis using microscopic examination suggested that the removal process of the two azo dyes is different. Removal of Sudan I was through biosorption, since it easily assembled and adsorbed on the surface of zoogloea due to its insolubility, while AO7 was biodegraded incompletely and bioconverted, the AO7 molecule was decomposed to benzene series and inorganic ions, since it could reach the interior area of zoogloea due to the low oxidation-reduction potential conditions and corresponding anaerobic microorganisms. The transformation of NH_3_-N, SO_4_^2−^ together with the presence of tryptophan-like components confirm that AO7 can be decomposed to non-toxic products in an aerobic bioreactor. This study provides a theoretical basis for the use of biosorption or biodegradation mechanisms for the treatment of different azo dyes in wastewater.

## 1. Introduction

Synthetic dyes are extensively used by various industries such as the textile industry, leather tanning industry, food technology, paper printing, and in photo-electrochemical cells [1,2]. Large scale production and the widespread application of synthetic dyes lead to severe contamination of surface and groundwater sources in the vicinity of dye industries [2]. Particularly, azo dyes, which are the largest class of synthetic aromatic compounds containing one or more azo groups [3], exhibit considerable structural diversity [1]. Untreated or partially treated azo dye effluent in water bodies can cause severe environmental or human health hazards [2]. Several physical (e.g., adsorption, coagulation, and membrane filtration), chemical (e.g., chemical oxidation, photo-catalytic, electrolysis, and Fenton reagent), and biological (biosorption, and enzymatic degradation) methods have been proposed for the treatment of dye wastewater [4]. Among them, microbial degradation of dyes has received significant research attention [5] because it is an eco-friendly and efficient treatment process [1,4,6]. Several microorganisms such as bacteria, fungi, yeast, and algae can degrade and even completely eliminate many dyes under certain environmental conditions [3,5,7].

A number of fungal strains such as white rot fungus were found to have the ability to decolorize dye wastewater [8], while some common fungi strains have the capacity of producing a wide variety of extracellular enzymes for azo dye decomposition [1]. Availability of suitable practical equipment for using fungal biomass is still at the research stage because of the harsh culture condition [8]. Algae in stabilization ponds can also play a significant role in degradation of azo dyes. However, it is necessary to isolate new strains or adapt existing ones for the decomposition of dyes [1]. In this context, anaerobic-aerobic biological methods appear to be appropriate for the degradation of dye-containing wastewater, because the –N=N– azo bond can be easily broken under anaerobic conditions and residual substances can further degrade under aerobic conditions [9]. However, aromatic amines produced by anaerobic process may decrease the microbial activity [10,11,12,13]. Hence, the restraint of noxious product or rapid degradation of aromatic amines should be considered in any microbial treatment process [3].

Most current studies on azo dye degradation focus on photochemical degradation methods. To our knowledge, only a few studies have investigated the biodegradation of azo dye in aerobic bioreactors. Although the effective mineralization of azo dye (Acid Orange 7) has been demonstrated in a pilot-scale vertical flow constructed wetland under aerobic condition, its degradation process involved coexistence of microbial and plant enzymes along with different oxidative conditions [14]. In the context of avoiding the toxic aromatic amine products, and shortening the treatment process, the aim of this paper was to study the biodegradation performance and to characterize different water-soluble azo dyes under pure aerobic conditions.

## 2. Materials and Methods

### 2.1. Experimental Setup and Bioreactor Operation

Three parallel units of aerobic bioreactors, secondary sedimentation banks, raw water inlet system, outlet system, recycle sludge system, air compressor system, and temperature control system were set up for the experiments. Aerobic bioreactors with a working volume of 4 L, an inner diameter of 120 mm and a height of 450 mm were selected for continuous stirred-bank operation.

Acid Orange 7 (sodium 4-[(2-hydroxy-1-naphthyl)azo]benzenesulphonate; CAS: 633-96-5; Aladdin, Shanghai, China) and Sudan I (1-phenylazo-2-naphthol; CAS: 842-07-9; Aladdin) (Figure S1) were selected to investigate the dye removal process in aerobic bioreactors. Both azo dyes have similar chemical construction with significantly different solubility (Table S1 in the Supplementary Materials). Similarly, glucose was used for comparison as it easily biodegrades compared to azo dyes.

Synthetic wastewater was fed into three reactors: Reactor A (RA), Reactor B (RB) and Reactor C (RC) in a downward direction at a pre-determined flow rate using a three-channel peristaltic pump to maintain equal flow. The primary composition of the inffluent from the three reactors is summarized in Table 1. The major component in the influent of RA, RB and RC reactors was glucose, Sudan I and Acid Orange 7, respectively. To increase the solubility, Sudan I was suspended in water, heated in a water bath at about 60 °C, and dissolved using ultrasonic method by sustaining for 0.5 h.

An air pump was used for continuous aeration through a porous stone air diffuser placed on the bottom of the bioreactors. During reactor operation, the oxygen concentration was maintained between 2 and 5 mg/L by adjusting the gas rotameter scale and by monitoring dissolved oxygen (DO) using the DO meter. The effluent wastewater was sampled every 24 h at the outlets. The bioreactors were operated at a steady temperature maintained by using a sheathed heater (Table S2 in the Supplementary Materials).

### 2.2. Analytical Methods

Influent and effluent samples were collected every day and immediately stored at 4 °C. The chemical oxygen demand (COD) and chromaticity in influent and effluent were determined according to the Standard Methods [15]. In the reactors, DO was measured with a fluorescence without membrane dissolved oxygen analyzer. Redox potential (ORP) was determined using compound gel filling electrode, the corresponding ORP can be described by the Nernst equation [16]:

(1) $$\mathrm{AO}7+O_{2}+H_{2}O=S_{1}+S_{2}+X$$

(2) $$E_{\mathrm{ORP}}=E_{\mathrm{ORP}}^{0}+0.0592\ln\left[\left({\mathrm{AO}}_{7}\right)\left(O_{2}\right)/\left(S_{1}\right)\left(S_{2}\right)\left(X\right)\right]$$

AO7: Acid Orange 7, S_1_: organic product, S_2_: primary inorganic product, X: other product.

Conductivity was measured by the standard electrode. Mixed liquid suspended solids (MLSS) was used to assess active sludge character and was measured by heating the mud mixture to constant weight in an oven at 105 °C followed by microscopic examination by optical microscope.

### 2.3. RRF (Relative Removal Factor) Analysis

As evident from the composition of the effluent (Table 1), COD was mainly contributed by starch (glucose) and azo dye. To determine the actual amount of azo dye removed from COD, the COD change in RA was compared with the other reactors. The COD of starch from inlet could be obtained by the theory of conversion. The relative removal factor (RRF) of azo dye from RB and RC was used to determine the actual removal of azo dye and was calculated using the following equation:

(3) $$\mathrm{RRF}=\left[\left({\mathrm{COD}}_{\mathrm{ea}}-{\mathrm{COD}}_{\mathrm{es}}\right)/\left({\mathrm{COD}}_{i}-500\right)\right]\times 100\%$$

where COD_ea_ is the effluent COD of RB or RC (mg/L), COD_es_ is the effluent COD of RA (mg/L), COD_i_ is the influent COD (mg/L), and 500 refers to the theoretical COD of starch from effluent (mg/L).

### 2.4. EPS (Extracellular Polymeric Substances) Extraction and Analysis

Mud mixtures of 0.015 L from aerobic bioreactors were centrifuged at 3500 rev/min for 10 min at room temperature. After discarding the supernatant, the sodium chloride solution of 0.9% (w/w) was added and the sludge pellets were resuspended. Similar procedure was followed three times to wash the sludge. Then, the samples were heated in water bath at 80 °C for 45 min, and then cooled to room temperature, before centrifuging at 5000 rev/min for 10 min. To obtain EPS, supernatants were filtered through 0.45 μm acetate cellulose membranes. The EPS concentration can be characterized by polysaccharides, protein and humic acids. The protein concentrations of EPS were measured by the modified Lowry method as described previously [17]. The polysaccharide concentrations of EPS were measured by the anthrone-sulfuric method as described previously [18,19].

### 2.5. EEM (Excitation Emission Matrix) Fluorescence Spectra

EEM fluorescence spectra were obtained using a Hitachi F-7000 fluorescence spectrophotometer (Hitachi Inc., Tokyo, Japan) equipped with a 150 W Xe-arc lamp at a PMT voltage of 700 V. Excitation and emission slit bandwidths were maintained at 10 nm and the scanning speed was set at 2400 nm/min with sampling intervals of 5 nm and 1 nm on excitation (Ex) and emission (Em) modes, respectively. The scanning field was set at emission spectra between 280 nm and 550 nm, whereas the excitation spectra were set from 200 nm to 450 nm. The spectrum of double distilled water was recorded as the blank, as the samples were diluted by double distilled water by 10 folds. Since EEM spectra were not used for quantitative analysis in this study, fluorescence intensities were not normalized and were presented with color variations. The peak Ex/Em pairs were picked with Hitachi FL Solutions software (Hitachi Inc., Tokyo, Japan).

## 3. Results

### 3.1. COD Removal Performance

The COD level, which would characterize the azo dye removal when compared with glucose, could reflect the organic matter of co-substrate, nutrient and azo dye in influent and effluent [20].

**Figure 1:**
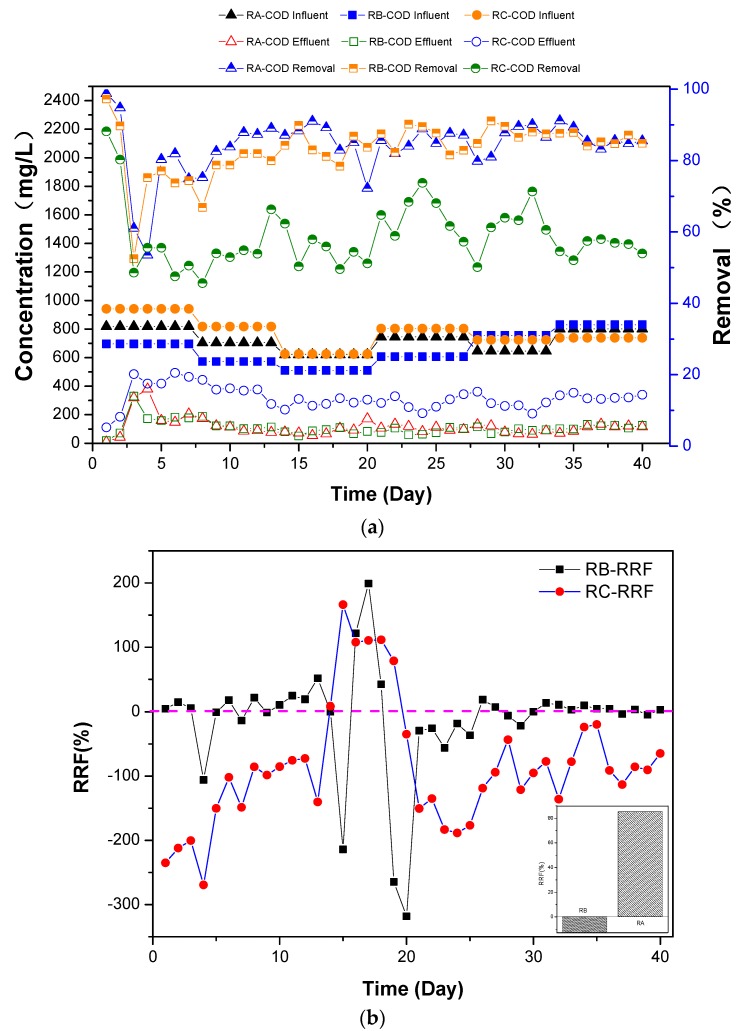
(**a**) COD (chemical oxygen demand) concentration and removal in the Reactor A (RA), Reactor B (RB) and Reactor C (RC); (**b**) RRF (relative removal factor) analysis of RB and RC.

At the beginning of the three days operation, COD removal efficiency in all bioreactors dropped drastically because of maladjustment of the primeval aerobic microorganisms. Then, the COD removal efficiency improved to varying degrees for the three bioreactors. The RA and RB reactors showed about 80% COD removal efficiency after 10 days of operation and acclimation, while the RC bioreactor achieved only about 60% COD removal efficiency (Figure 1a).

RRF analysis can be used to better evaluate the removal of azo dye. From RRF equation, the easily degradable substance such as starch in the inlet of RB and RC reactors were deducted from theoretical COD value, while the COD at the outlet from RB and RC reactors were compared with RA reactor. When the RRF value approached to zero, the azo dye removal efficiency followed the readily biodegradable substance behavior as in the RA reactor. The RC-RRF value deviated from zero and was positive, and their average reached +85.3% after 10 days of operation and acclimation. However, the RB-RRF value approached to zero, and the mean RRF value was −12.7% for the same period (Figure 1b). This indicates that Sudan I azo dye had high removal efficiency similar to glucose in the RA reactor and can even achieve complete removal, while AO7 was incompletely degraded. 

### 3.2. Sudan I Removal Performances

**Figure 2:**
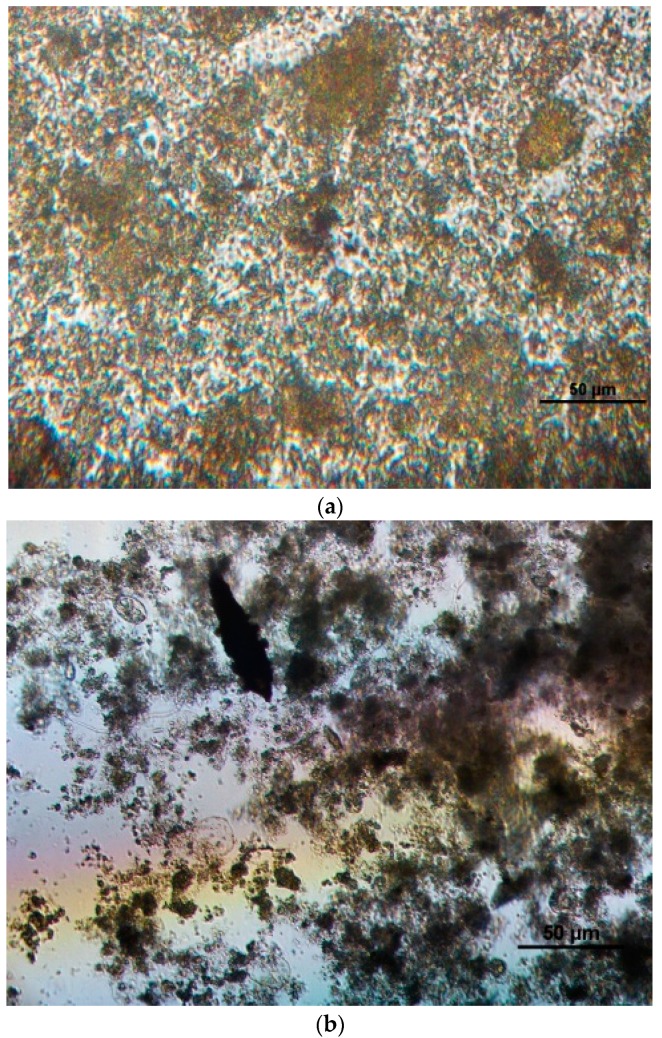
Image ofactivated sludge phase detected by optical microscope (100 folds) from (**a**) RB and (**b**) RC reactors.

Aerobic biomass biosorption processes can be investigated through light microscopy techniques. It can be seen that little red rot is distributed on the surface of zooglea (Figure 2a), whereas it did not appear beside zooglea (Figure 2b).

This suggests that Sudan I, which is an insoluble azo dye, could be easily aggregated as tiny particulate matters attached to zooglea. Due to its good solubility, it was difficult for AO7 to accumulate on the surface of zooglea. Moreover, from the above section, it was found that Sudan I was not decomposed completely in the RB reactor. Therefore, it can be concluded that high Sudan I removal efficiency was due to the biosorption, not because of the biodegradation and mineralization.

### 3.3. AO7 Degradation

From the analysis of RC reactor effluent samples at different stages (Table S3 in the Supplementary Materials), it was found that sulfate (SO_4_^2−^) and ammonia-N (NH_3_-N) levels increased at the 30th day, by 60 mg/L and 1 mg/L, respectively, while nitrate-N (NO_3_^−^) and nitrite-N (NO_2_^−^) were not detected. This result demonstrates that the SO_4_^2−^ was released to the liquid phase from the AO7 molecules, while NH_3_-N was also released from the azo structures in the molecule by biotransformation. The increased value of SO_4_^2−^ and presence of NH_3_-N (the presence of NH_3_-N in inlet was probably from the impurities of the azo dye agentia) provide strong evidence that the azo dye AO7 is being degraded. Since the transformation of NH_3_-N from RB reactor is not significant, it can be concluded that Sudan I molecule was not removed completely [21,22].

**Figure 3:**
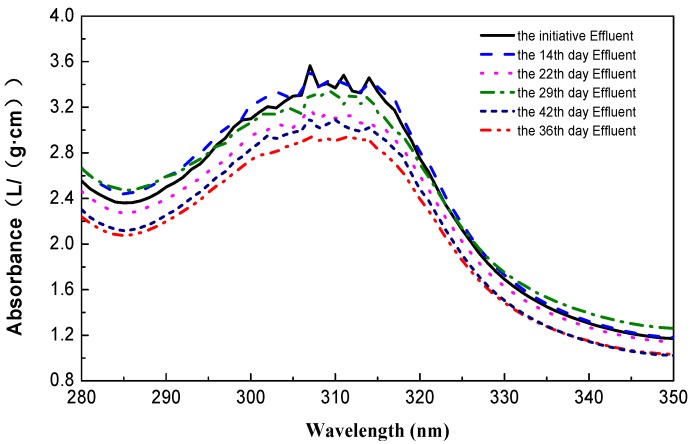
Absorbance of effluent from RC reactor by fall wave scanning at different stages.

Azo dyes, as groups of electron-deficient xenobiotic compounds [13], are reported recalcitrant against aerobic bacterial degradation, and aerobic processing of azo dyes has been proven ineffective [23,24,25]. However, from the above section, actual removal of AO7 azo dye was achieved (Figure 1b). If AO7 in the RC reactor influent were completely converted to the COD, the average value could reach approximate 275 mg/L (COD_i_ − 500). The mean residual COD, i.e., COD_ea_ − COD_es_, of AO7 from RC reactor effluent was about 200 mg/L (Figure 1b, Equation (3)), indicating that AO7 could partially be mineralized in aerobic conditions. As can be seen in Figure 3, the absorbance gradually drops with the increasing days of acclimation, in particular, the prime absorption peaks of inchoate effluent disappeared after three weeks of operation. This indicates that the concentration of azo dye decreased and the structure of AO7 molecules was also changed. Further, this shows that the azo dyes such as AO7 could biodegrade in an aerobic environment.

**Figure 4:**
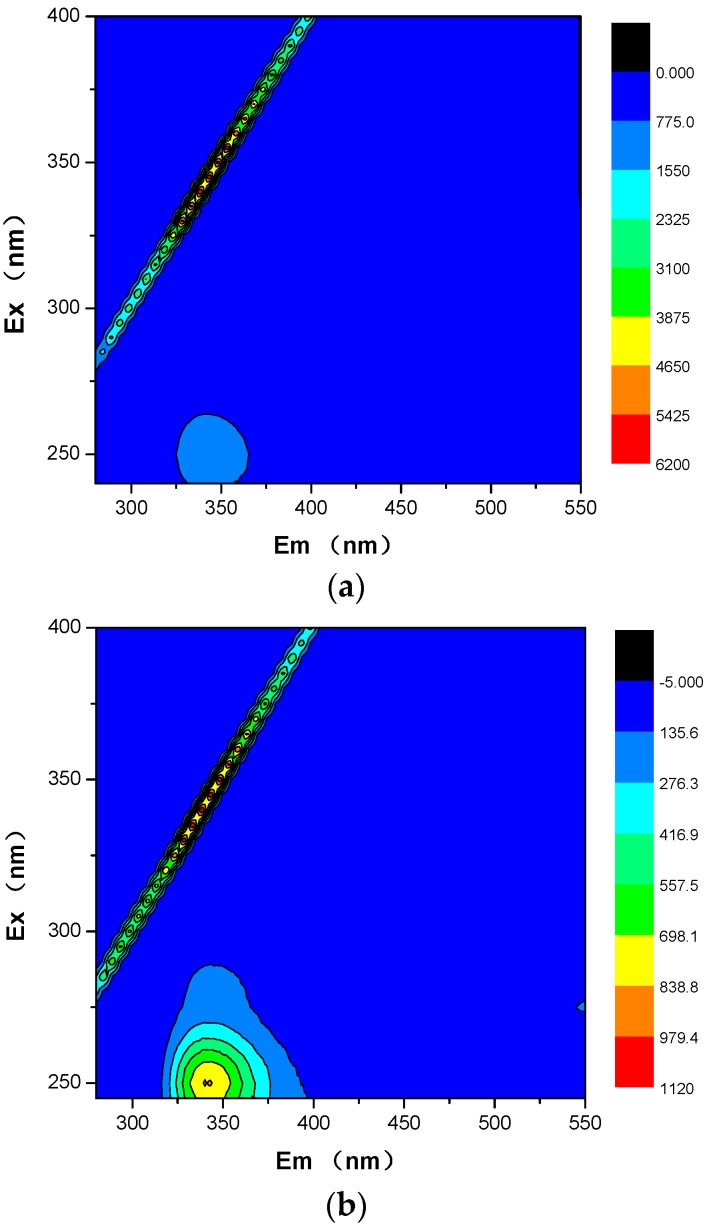
EEM (excitation-emission-matrix) fluorescence spectra of (**a**) RC reactor effluent at two weeks, and (**b**) in a month.

The composition of dissolved organic matter (DOM) from biotreatment effluent could be characterized by three-dimensional excitation-emission-matrix (EEM) fluorescence spectrometry [14,26]. In this study, three-dimensional EEM spectroscopy was applied for characterizing the DOM of RC reactor effluent, which was collected for two weeks and a month. The main peaks at excitation/emission wavelengths (Ex/Em) of 250/340 nm simultaneously existed, and were intensity peak describing tryptophan-like component in Figure 4 [27,28]. The result indicates that tryptophan-like component in RC reactor increased with acclimatization days, though they were not found in the RB reactor (Figure S2 in the Supplementary Materials). As a microorganism metabolism product, tryptophan-like substance demonstrates that the AO7 molecule was decomposed in the aerobic bioreactor.

### 3.4. Discussion

The COD removal rates of the three reactors indicate that the organic contaminants could be removed to varying degrees by active sludge (Figure 1a,b), and that aerobic biochemical treatment process can use different mechanisms to remove dye molecules with azo structures. RA achieved a desirable COD removal rate of about 80%, which was higher than 60% for the electrochemical degradation process based on the Fdoped-tin oxide glass [29]. The aerobic degradation of Sudan I seems more efficient and economical. Previous studies on azo dye removal using fungi proposed various mechanisms such as biosorption, bioaccumulation and biodegradation. Fungi are preferred for dye removal due to their high cell-to-surface ratio and the extra-cellular nature [4]. *Aspergillus flavus*, which is a common fungal biomass, was used to remove Remazol Black B dye (RBB) [30]. Several research studies indicated that biosorption by fungi is effective for the removal of azo dyes, though their cultivation generally requires harsh environment in biochemical incubators [1]. The zoogloea consist of microbial colonies, which are characterized by functionally specialized cell components, and this symbiont cells are capable of working together for contaminant degradation [31]. Moreover, biosorption involves a number of complex processes such as physical and chemical adsorption, electrostatic interaction, ion exchange, complexation, chelation, and microprecipitation, and is an effective technology to remove dye molecules from dilute solutions [32]. From above results, Sudan I azo dye removal mechanism was found to be biosorption. Because of insoluble and agminate character of Sudan I, they were gathered readily and adsorbed by the zooglea.

The lower removal rate of the RC reactor (Figure 1b) implies that the azo dye AO7 resists biodegradation directly under aerobic conditions, though it can be partly degraded and its molecular structure could be changed (Figure 1b) or decomposed (Figure 3). This contradicts the previous general opinion on azo dye biodegradation, that is, it generally occurs through anaerobic and aerobic processes, involving reductive cleavage of the azo linkages resulting in the formation of aromatic amines [33] (Figure S3 in the Supplementary Materials). However, after nearly a month of cultivation in this laboratory-scale experiment, tryptophan-like component in the RC reactor effluent (Figure 4b) indicated that the –N=N– bond of AO7 was decomposed and azo linkage cleaved in the aerobic bioreactor. Further, the transformation of SO_4_^2−^ and NH_3_-N was confirmed as the cleavage of AO7.

**Figure 5:**
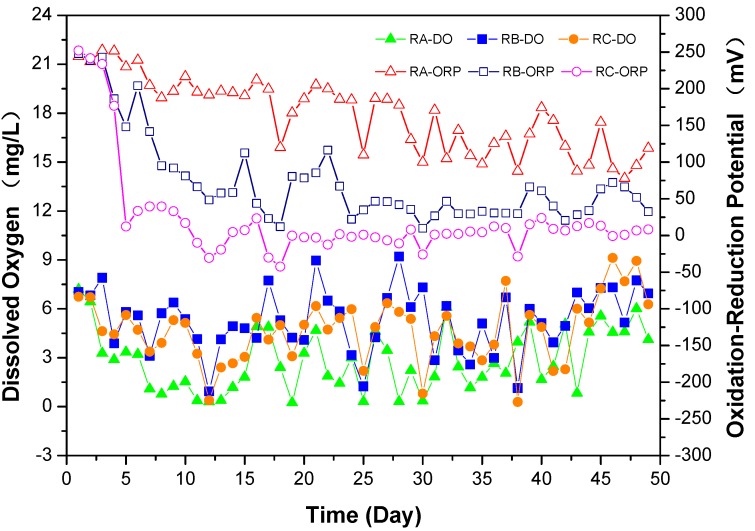
Change of dissolved oxygen and oxidation reduction potential in three reactors.

According to Equations (1) and (2), as [O_2_] is a constant, the $E_{\mathrm{ORP}}^{0}$ is a definite value, the concentration of [S_1_], [S_2_] and [X] were higher than the [AO7] because of biodegradation with the increase in cultivation time. At the 5th day of operation, NH_3_-N and SO_4_^2−^ enhanced (Table S3 in the Supplementary Materials), while the ORP sharply dropped (Figure 5), which is in accordance with the Nernst equation. Although there is enough oxygen concentration, NH_3_-N cannot undergo an amine oxidation process under aerobic conditions due the low ORP condition. 

According to the results obtained, although their molecule structure was similar, the aerobic biodegradation process and the mechanism of Sudan I and AO7 degradation were different. The sulfonic acid group affected the water solubility of dyes and the biodegradation mechanism. Since AO7 dyes were dissolved in the water, they could more easily approach the interior area of zoogloea than Sudan I and be reductively decomposed to sulfonated aromatic amines and 1-amino 2-naphthol [33] by anaerobic bacteria under partially anaerobic conditions. 1-Amino 2-naphthol was released from the interior anaerobic area to the exterior aerobic area and decomposed to NH_3_-N and benzene products, which could be further degraded [34,35,36,37,38]. Then, the sulfonic acid group of the sulfonated aromatic amine was reduced to SO_4_^2−^ and 4-aminophenol in the interior anaerobic area, while SO_4_^2−^ was released to liquid phase because of the insufficient low ORP atmosphere. 4-Aminophenol was transferred to the exterior aerobic area and was further degraded and mineralized by aerobic microbes and the corresponding enzymes. In all the above processes, NH_3_-N was hardly oxidized due to the low ORP rather than only dissolved oxygen.

The COD removal of Sudan I could reach more than 85% of the glucose level by day 40 because of the insolubility of the azo dye which could be adsorbed on the zoogloea. Due to the low ORP of the interior area, the AO7 molecule could be decomposed to sulfonated aromatic amines and 1-amino 2-naphthol smoothly, yielding compounds could be mineralized at the end [39]. However, since the intermediate products, sulfonated aromatic amines, are of xenobiotic character [25,39,40], they inhibited the AO7 molecule decomposition until sulfonated aromatic amines were bio-converted in the interior anaerobic area. This is the reason for AO7 being resistant to biodegradation. Furthermore, aromatic amine products from AO7 can be gradually generated in the interior anaerobic area because they were not detected in the RC reactor. The toxicity caused by aromatic amines could be avoided.

## 4. Conclusions

The biodegradation performance and characteristics of Sudan I and AO7 were investigated under aerobic conditions in parallel continuous-flow mixed stirred reactors. After 40 days of reaction, the COD removal rate of the Sudan I matrix reactor (RB) reached 85%, close to that of the glucose matrix reactor (RA). The removal rate of COD was less than 60% in the reactor containing AO7. High Sudan I removal efficiency that is closer to that of the glucose is due to biosorption, not due to biodegradation and mineralization. The transformation of NH_3_-N, SO_4_^2−^ and the presence of tryptophan-like components confirm that AO7 can be decomposed to benzene series compounds and inorganic ions in an aerobic bioreactor. It can be concluded that the biosorption or biodegradation phenomena are different for dyes with different water-solubility, and is similar for azo dyes with similar molecular structures.

## Figures and Tables

**Table 1:** Major composition of inffluent from reactors.

Trial	Reactor	Starch (mg L^−1^)	Sodium Sulfate (mg L^−1^)	Glucose (mg L^−1^)	Sudan I (m mol L^−1^)	Acid Orange 7 (m mol L^−1^)
1	Reactor A (RA)	500 ± 55	1250 ± 11	230 ± 50	--	--
2	Reactor B (RB)	500 ± 50	1250 ± 13	--	0.75 ± 0.6	--
3	Reactor C (RC)	500 ± 53	1250 ± 10	--	--	0.75 ± 0.2

## Supplementary Materials

The following are available online at www.mdpi.com/1660-4601/15/1/35/s1, Figure S1. Chemical structure of (a) Acid Orange 7 and (b) Sudan I, Figure S2. (a) EEM fluorescence spectra of effluent from RB reactor after two weeks of operation. (b) EEM fluorescence spectra of effluent from RB reactor a month of operation, Figure S3. Cleavage pathway of AO7 under traditional anaerobic condition, Table S1. Physicochemical property of Sudan I and AO7, Table S2. Experimental conditions, Table S3. Concentration of ion at RC reactor inlet and outlet.
