# Supplementary material for: Aerobic Biodegradation Characteristic of Different Water-Soluble Azo Dyes
Source: Int J Environ Res Public Health. 2017 Dec 26;15(1):35. doi: 10.3390/ijerph15010035 (PMC5800135; doi:10.3390/ijerph15010035)
Supplement: Supplementary file 1 [file ijerph-15-00035-s001.pdf]

Supplementary Material

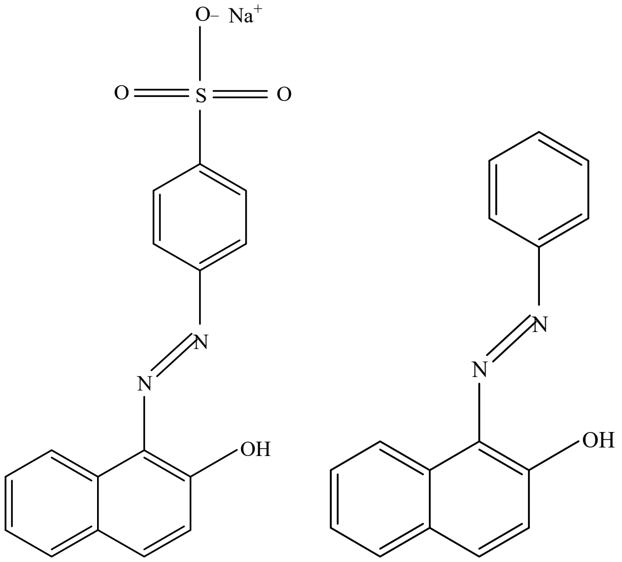

(**a**) (**b**)

Figure S1. Chemical structure of (**a**) Acid Orange 7 and (**b**) Sudan I.

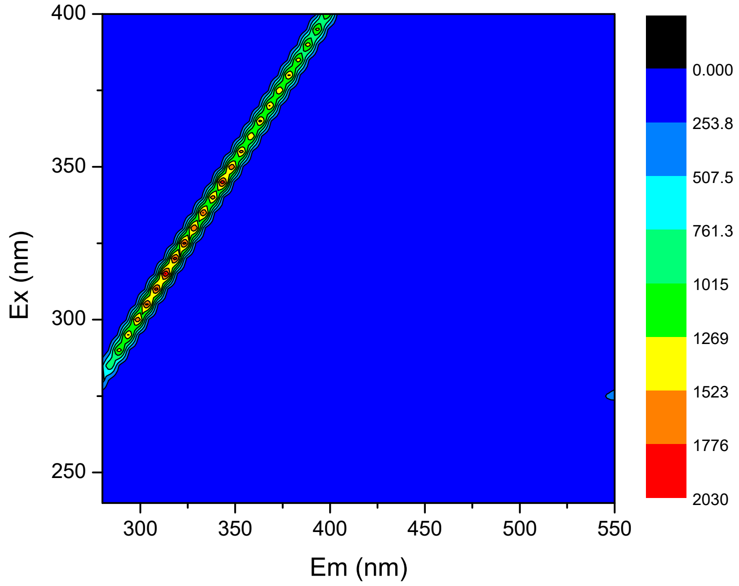

(**a**)

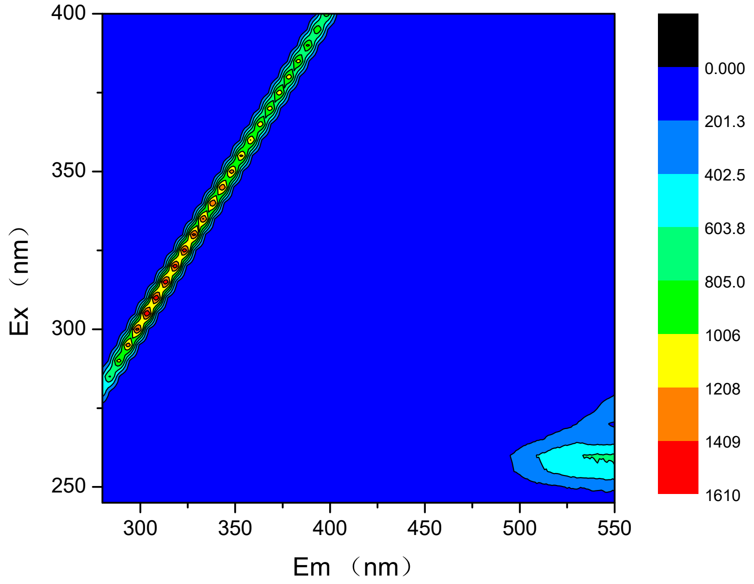

(**b**)

Figure S2. (**a**) EEM fluorescence spectra of effluent from RB reactor after two weeks of operation. (**b**) EEM fluorescence spectra of effluent from RB reactor a month of operation.

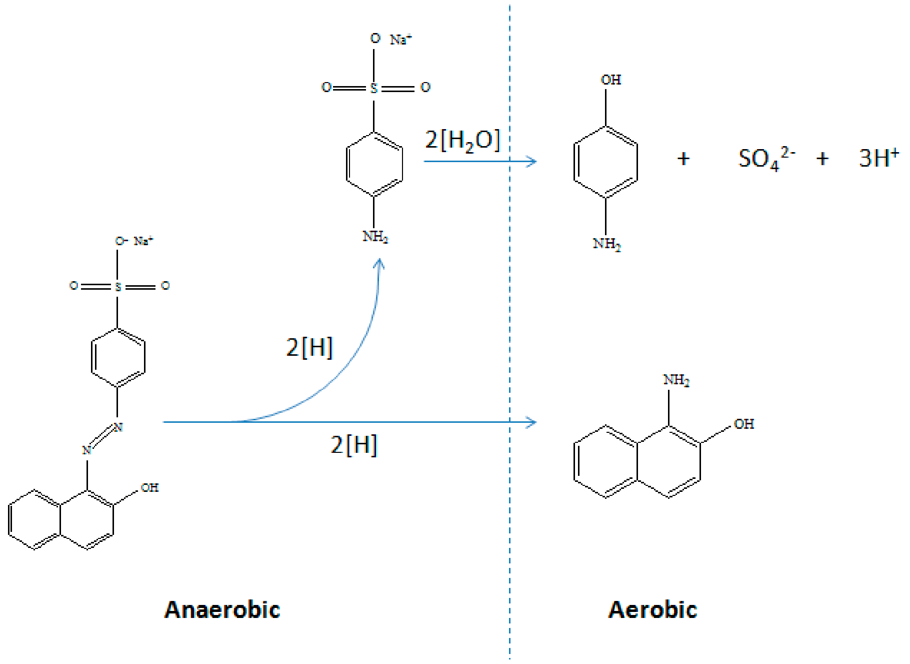

Figure S3. Cleavage pathway of AO7 under traditional anaerobic condition.

Table S1. Physicochemical property of Sudan I and AO7.

Trial	Azo Dye	Molecular Weight	Soluble ($g \cdot L^{-1}$)	Molecular Formula
1	Sudan I	248.28	0.5 (30°C)	$C_{16}H_{12}N_2O$
2	Acid orange 7	350.32	116 (30°C)	$C_{16}H_{11}N_2NaO_4S$

Table S2. Experimental conditions.

Trial	Reactor	Temperature (°C)	Liquid Flow ($mL \cdot min^{-1}$)	Air Flow ($mL \cdot min^{-1}$)	MLSS ($mg \cdot L^{-1}$)
1	RA	19 ± 3	2.46 ± 0.14	65 ± 28	3551 ± 822
2	RB	19 ± 3	2.40 ± 0.18	39 ± 20	2378 ± 1035
3	RC	19 ± 3	2.34 ± 0.16	47 ± 22	2329 ± 917

Table S3. Concentration of ion at RC reactor inlet and outlet.

Trial	Sample	NO_2^- ($mg \cdot L^{-1}$)	NO_3^- ($mg \cdot L^{-1}$)	NH_3-N ($mg \cdot L^{-1}$)	SO_4^{2-} ($mg \cdot L^{-1}$)	T.D.S ($\mu s \cdot cm^{-1}$)
1	Inlet	0	0	0.21	682	1553
2	The 3th day outlet	0	0.12	0.13	684	1552
3	The 5th day outlet	0	0	0.46	709	1555
4	The 30th day outlet	0	0	1.22	740	1552
